# Prolonged COVID-19 in a Multiple Sclerosis Patient Treated With Rituximab

**DOI:** 10.7759/cureus.32523

**Published:** 2022-12-14

**Authors:** Nikos Kintrilis, Charilaos P Gkinos, Iosif Galinos

**Affiliations:** 1 Department of Physiology, National and Kapodistrian University of Athens, Athens, GRC; 2 Second Department of Internal Medicine, 401 General Military Hospital of Athens, Athens, GRC; 3 Infectious Diseases Unit, 401 General Military Hospital of Athens, Athens, GRC

**Keywords:** long covid, coronavirus disease, novel coronavirus, vaccine, humoral immunity, immunosuppression, rituximab, remdesivir, covid-19, sars-cov-2

## Abstract

Immunocompromised patients commonly present prolonged viral shedding of the novel coronavirus SARS-CoV-2, detected through reverse transcriptase-polymerase chain reaction (RT-PCR) in nasopharyngeal or oropharyngeal swabs. The detection and estimation of the viral load in patients with COVID-19 is of utmost importance, not only for the effective isolation of the patient but also from a therapeutic point of view. In the current study, we present the case of an immunocompromised patient receiving rituximab infusions for the treatment of multiple sclerosis who exhibited COVID-19 clinical symptomatology for an extended period of time along with prolonged viral shedding while at the same time being unable to organize sufficient humoral immunity. Despite being fully vaccinated and having suffered symptomatic SARS-CoV-2 infections, antibodies against the virus remained undetected. Clinical relapse of his symptoms led to the trialing of a multitude of therapeutic interventions in order to combat the disease, with an extended remdesivir regimen proving the most efficacious in the alleviation of his symptoms. This case demonstrates how immunocompromised COVID-19 patients should be regarded under a different scope when it comes to the diagnosis, management, and resolution of their SARS-CoV-2 infection.

## Introduction

The severe acute respiratory syndrome coronavirus 2 (SARS-CoV-2) novel coronavirus has affected the globe for three years now and was first described in Wuhan, China, in late 2019 [1]. Within the Greek borders, the first case of coronavirus disease-19 (COVID-19) was reported on February 26, 2020, and since then, more than 5.2 million total cases have been noted, costing the lives of almost 34,000 people [2]. The disease can present with a plethora of clinical manifestations, ranging from asymptomatic infection to severe acute respiratory distress syndrome with multiple complications and even death [3]. Viral shedding refers to the period during which a SARS-CoV-2-infected patient tests positive with a nasopharyngeal swab, sputum sample, or endotracheal aspirate sample, with detection of the virus usually quantified by RT-PCR amplification. Even though viral shedding times do not seem to differ significantly based on the severity of the disease, a more severe course of the disease may predict prolonged viral shedding [4]. Although RT-PCR amplification techniques are nowadays routinely performed on an everyday basis, since the beginning of the pandemic, it has been impossible to correlate the positivity of the test with the viral load [5]. Initial reports suggested infectivity periods ranging from 6.5-9.5 days in asymptomatic patients [6], while newer variants presented a lower infectivity dynamic with subsequent changes in the isolation protocols [7]. In immunocompromised patients, viral shedding periods may differ, making it more difficult to distinguish between symptom persistence and clinical relapse of infection [8-10]. As for patients on regimens including rituximab alone or in combination with other immunosuppressive drugs, there have been reports of persistent viral shedding, with implications for the requirement for a special approach [11,12]. In the present study, we describe the case of an immunocompromised multiple sclerosis patient receiving rituximab who exhibited a prolonged COVID-19 disease phase, including relapse of his symptoms, prolonged viral shedding, and an inability to build immunity against the virus despite both vaccination and infection.

## Case presentation

A 49-year-old male with a personal history of multiple sclerosis on a therapeutic regimen of infusions of 700 mg rituximab once every six months presented to the emergency department (ED) of our hospital due to fever for the preceding two days, along with dry cough, arthralgias, and general malaise. Apart from his neurological disease, the patient had no other medical conditions, and he denied using tobacco, alcohol, or recreational drugs. He received his last rituximab infusion approximately one month ago. Apart from rituximab, he was receiving mirtazapine and a vitamin D supplement. The patient was fully vaccinated against SARS-CoV-2 with three Pfizer vaccine doses, the last of which had taken place approximately four months prior to his ED presentation. At presentation, his vital signs showed a fever of 38.1°C, a respiratory rate (RR) of 16 breaths per minute for oxygen saturation (SaO2) of 97% on room air, a blood pressure (BP) of 150/95 mmHg, and a heart rate of 91 beats per minute (bpm). A rapid antigen test and a nasopharyngeal PCR SARS-CoV-2 test were performed upon his arrival, both of which were positive. Blood was drawn for a complete blood count, biochemical profile, and arterial blood gases, with the results being depicted in Table 1.

**Table 1:** Complete laboratory examinations at the ED on the first visit and subsequent relapses. pO2: partial pressure of oxygen; pCO2: partial pressure of carbon dioxide; HCO3: bicarbonate; SaO2: oxygen saturation

Examination	First visit (Day one)	First relapse (Day 15)	Second relapse (Day 43)	Laboratory reference range
White blood cells (K/μL)	8.9	5.8	6.7	4.0-10.8
Neutrophils (%)	69	76	89	40-75
Lymphocytes (%)	23	15	8	20-45
Monocytes (%)	8	9	3	2-10
Red blood cells (M/μL)	4.1	4.4	4.2	4.5-6.1
Hemoglobin (g/dL)	12.5	13.6	12.9	13.5-17.9
Hematocrit (%)	36.6	38.9	37.5	40.0-52.0
Platelets	152	186	245	150-440
Potassium (mmol/L)	3.9	4.10	4.90	3.60-5.00
Sodium (mmol/L)	142	130	137	135-150
Glucose (mg/dL)	74	98	185	70-110
Urea (mg/dL)	39	20.0	34	20.0-50.0
Lactate dehydrogenase (U/L)	583	241	818	120-246
Creatine phosphokinase (U/L)	48	543	71	30-170
Alkaline phosphatase (U/L)	79	56	116	38-126
γ-glutamyltransferase (U/L)	170	43.0	180	8.0-78.0
Aspartate aminotransferase (U/L)	37	34	140	15-59
Alanine aminotransferase (U/L)	85	26	255	10-72
Total bilirubin (mg/dL)	0.40	0.50	0.20	0.20-1.30
C-reactive protein (mg/L)	4.9	21.0	75.2	0.0-5.0
Ferritin (mg/L)	878.0	206.5	2412.0	20.0-280.0
Procalcitonin (ng/mL)	0.03	0.03	0.09	<0.10
International normalized ratio	0.89	0.88	0.85	0.85-1.15
D-dimer (μg/L)	411.30	538.50	1066.00	0.00-500.00
Arterial blood gas on air				
pH	7.43	7.44	7.46	
pO_2 _(mmHg)	77.7	65.3	56.1	
pCO_2 _(mmHg)	36.4	34.5	30.9	
HCO_3 _(mmol/L)	24.0	23.2	21.1	
SaO_2_ (%)	96.1	93.8	89.7	
Urinalysis	negative	negative	negative	

The patient was admitted to the infectious diseases unit of our hospital, where he was placed on a remdesivir regimen of 200 mg on day one and 100 mg on subsequent days, intravenous dexamethasone 6 mg, and empirical antibiotic treatment with piperacillin-tazobactam 4.5 g three times daily and vancomycin 1 g twice daily. On the fifth day after his admission, the patient remained febrile, and various sets of aerobic and anaerobic blood cultures had been drawn without any pathogens being cultured. Due to concerns for hospital-acquired pneumonia (HAP), his antibiotic regimen was empirically adjusted with the addition of trimethoprim-sulfamethoxazole and caspofungin for pneumocystis carinii pneumonia (PCP) and fungal pathogen coverage, respectively. Although the patient remained afebrile for 24 hours, his fever came back, and all antibiotics were discontinued, with the patient receiving only remdesivir and cortisone therapy. At the same time, on the tenth day after his initial ED presentation, a quantitative measurement of the patient’s SARS-CoV-2 antibodies was negative (6.20 AU/mL, positive reference value ≥50.0 AU/mL). Cortisone therapy was changed to higher doses of methylprednisolone (40 mg twice daily) with a plan to taper over the next few weeks, and the patient remained afebrile for the next 48 hours with inflammatory markers subsiding. He was discharged on the 12th day, only to come back two days later with a new onset of pyrexia and worsening respiratory status, as depicted in Table 1.

**Figure 1:**
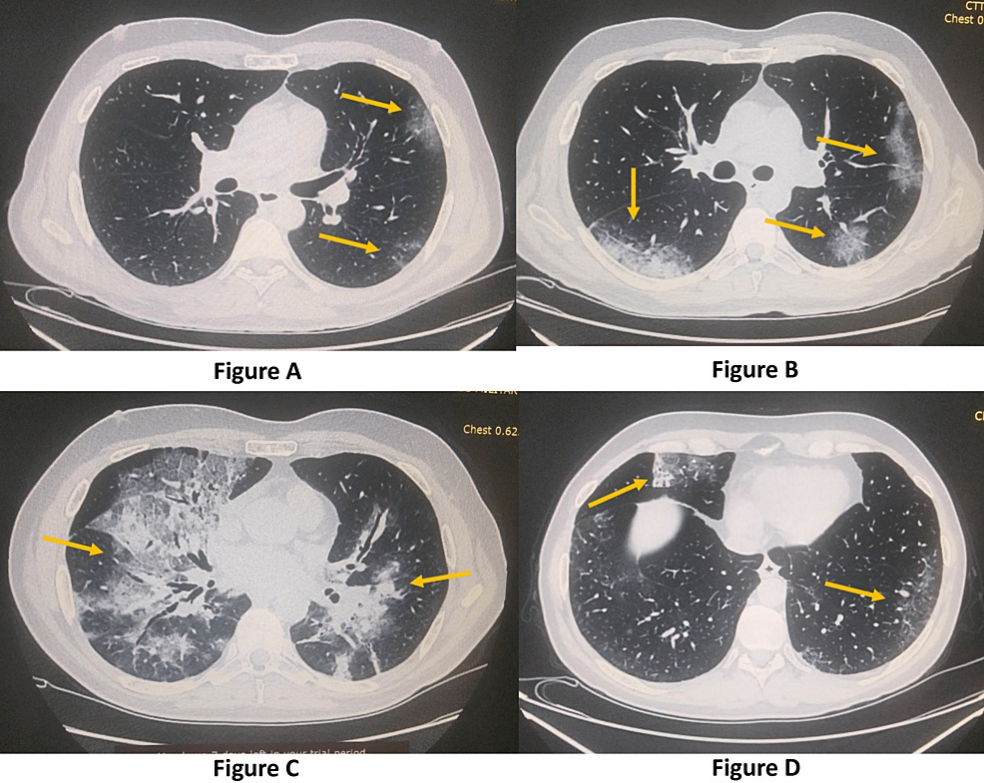
Chest computed tomography (CT) at various points of the disease. Figure 1A: Initial patient presentation at the ED and admission. Figure 1B: First disease relapse with mild radiographic worsening. Figure 1C: Second disease relapse with severe radiographic worsening. Figure 1D: Follow-up examination with dramatic radiographic improvement.

Laboratory examinations revealed elevated ferritin and lactate dehydrogenase (LDH) without other adverse prognostic factors (CRP, D-dimer, and lymphocytes were within normal range, and arterial blood gas revealed no hypoxemia). A computed tomography (CT) scan of the chest showed bilateral ground glass opacities compatible with COVID-19 pneumonia (Figure 1A). Remdesivir and intravenous methylprednisolone were reinstated; however, owing to a transient worsening of his respiratory status and the radiographic progression of his chest CT (Figure 1Β), a possible diagnosis of cryptogenic organizing pneumonia (COP) was suspected, and a bronchoscopy was performed. Examinations of bronchoalveolar lavage (BAL) revealed no signs of malignancy, infection, or inflammation, and BAL cultures were also negative, while SARS-CoV-2 PCR testing of BAL was positive. The patient improved clinically after ten days of treatment and was sent home once again. As soon as cortisone therapy was tapered down, 43 days after his initial presentation, the fever relapsed once more, and the patient returned for a third time to the ED, this time with much worse respiratory status and inflammatory markers. Radiological images were dramatically worse as well (Figure 1C). He was started on a new course of remdesivir (loading 200 mg and 100 mg daily thereafter), while he also received intravenous gamma-globulin (IVIG) this time to support his immunological response. His pyrexia lasted seven more days until he finally improved clinically, with all symptoms and laboratory findings finally subsiding. A new nasopharyngeal swab was collected, which once again revealed a positive PCR test, while a second quantitative IgG antibody test was negative. The patient was discharged and returned one week later for a follow-up examination, with his CT scan and breathing function having drastically improved (Figure 1D).

Nasopharyngeal swab specimens were collected for PCR testing every 14 days thereafter, with the patient expressing positivity for more than 120 days before finally becoming negative on day 127 after his initial presentation to the hospital. A third and final quantitative SARS-CoV-2 antibody test was negative, and the patient opted for a fourth vaccine dose, which was administered a few weeks later.

## Discussion

Prolonged SARS-CoV-2 viral shedding has recently been described in the literature in immunocompromised hosts, including patients on rituximab regimens. Individuals in this case presented with a varying degree of waxing and waning of their symptoms, while their disease commonly relapsed, leading to the administration of various therapeutic schemes to combat the disease [9, 13-15]. Optimal therapeutic measures for the population under rituximab treatment remain under investigation, with most authors trialing regimens combining immunotherapy to boost the immune reaction along with prolonged administration of classic antiviral medications such as remdesivir [16,17]. At the same time, vaccines approved for the disease so far have demonstrated reduced efficacy in immunocompromised individuals [18], with some patients benefiting from a further dose of the vaccine [19]. In rituximab-treated patients, SARS-CoV-2 vaccination elicited specific antibodies as long as peripheral B cells were able to repopulate after treatment, at least partially. The majority of patients receiving the vaccine, though, were able to build protective T cells regardless of their humoral immune response [20].

Furthermore, the use of high corticosteroid doses in the management of the present case needs to be highlighted. Dexamethasone use reduces mortality rates in hospitalized patients with severe COVID-19 who require supplemental oxygen support [21], but concerns about possible increased rates of hospital-associated infections were raised shortly after its approval as first-line treatment [22]. Of note, though, unlike other infections such as influenza, COVID-19 is complicated by superinfections at a much lower rate with corticosteroid use [23]. The benefit of high corticosteroid doses in already immunocompromised individuals as well as the mechanisms through which a possible benefit may be conferred remains a topic for further research.

What remains of utmost importance though is the fact that isolation protocols need to be adjusted accordingly for the group of immunocompromised patients. Effective isolation of affected individuals is mandatory not only for closer observation of their symptoms but also to ensure prophylaxis of their surrounding persons, carers, and even other immunocompromised patients that may exist in their environment. Given that there have been reports of extremely prolonged viral shedding times among immunocompromised individuals, even those experiencing milder clinical complaints [24], the Centers for Disease Control and Prevention (CDC) currently recommend self-isolating for at least 10 days and up to 20 days for immunocompromised individuals, whereas severely immunocompromised people should also implement a strategy of serial PCR testing and consultation with an infectious disease specialist [25].

## Conclusions

Concluding, our case regards an immunocompromised multiple sclerosis patient under a rituximab treatment regimen whose COVID-19 presented with prolonged clinical symptomatology along with extended persistence of viral shedding, as proven by repeated viral RT-PCR on nasopharyngeal specimen swabs. At the same time, the patient was incapable of eliciting protective SARS-CoV-2 antibodies even after completing a full vaccination series and suffering from the clinical syndrome. This case adds to the literature on COVID-19 disease in immunocompromised patients, especially the rituximab-treated population, highlighting the need for further research when it comes to these individuals’ hospitalization and drug regimens, isolation, and general management.

## References

[1] Zhu N, Zhang D, Wang W (2020). A novel coronavirus from patients with pneumonia in China, 2019. N Engl J Med.

[2] (2022). COVID-19 coronavirus pandemic. https://www.worldometers.info/coronavirus/.

[3] Esakandari H, Nabi-Afjadi M, Fakkari-Afjadi J, Farahmandian N, Miresmaeili SM, Bahreini E (2020). A comprehensive review of COVID-19 characteristics. Biol Proced Online.

[4] Munker D, Osterman A, Stubbe H (2021). Dynamics of SARS-CoV-2 shedding in the respiratory tract depends on the severity of disease in COVID-19 patients. Eur Respir J.

[5] Oran DP, Topol EJ (2021). The proportion of SARS-CoV-2 infections that are asymptomatic: a systematic review. Ann Intern Med.

[6] Byrne AW, McEvoy D, Collins AB (2020). Inferred duration of infectious period of SARS-CoV-2: rapid scoping review and analysis of available evidence for asymptomatic and symptomatic COVID-19 cases. BMJ Open.

[7] Keske Ş, Güney-Esken G, Vatansever C (2022). Duration of infectious shedding of SARS-CoV-2 Omicron variant and its relation with symptoms. Clin Microbiol Infect.

[8] Nakajima Y, Ogai A, Furukawa K (2021). Prolonged viral shedding of SARS-CoV-2 in an immunocompromised patient. J Infect Chemother.

[9] Leung WF, Chorlton S, Tyson J (2022). COVID-19 in an immunocompromised host: persistent shedding of viable SARS-CoV-2 and emergence of multiple mutations: a case report. Int J Infect Dis.

[10] Niyonkuru M, Pedersen RM, Assing K, Andersen TE, Skov MN, Johansen IS, Madsen LW (2021). Prolonged viral shedding of SARS-CoV-2 in two immunocompromised patients, a case report. BMC Infect Dis.

[11] Thornton CS, Huntley K, Berenger BM (2022). Prolonged SARS-CoV-2 infection following rituximab treatment: clinical course and response to therapeutic interventions correlated with quantitative viral cultures and cycle threshold values. Antimicrob Resist Infect Control.

[12] Arai T, Mukai S, Kazama R, Ogawa Y, Nishida K, Hatanaka K, Gohma I (2022). Persistent viral shedding of severe acute respiratory syndrome coronavirus 2 after treatment with bendamustine and rituximab: a case report. J Infect Chemother.

[13] Baang JH, Smith C, Mirabelli C (2021). Prolonged severe acute respiratory syndrome coronavirus 2 replication in an immunocompromised patient. J Infect Dis.

[14] Choi B, Choudhary MC, Regan J (2020). Persistence and evolution of SARS-CoV-2 in an immunocompromised host. N Engl J Med.

[15] Meiring S, Tempia S, Bhiman JN (2022). Prolonged shedding of severe acute respiratory syndrome coronavirus 2 (SARS-CoV-2) at high viral loads among hospitalized immunocompromised persons living with human immunodeficiency virus (HIV), South Africa. Clin Infect Dis.

[16] Furlan A, Forner G, Cipriani L, Vian E, Rigoli R, Gherlinzoni F, Scotton P (2021). COVID-19 in B cell-depleted patients after rituximab: a diagnostic and therapeutic challenge. Front Immunol.

[17] Rüfenacht S, Gantenbein P, Boggian K (2022). Remdesivir in coronavirus disease 2019 patients treated with anti-CD20 monoclonal antibodies: a case series. Infection.

[18] Gentile I, Schiano Moriello N (2022). COVID-19 prophylaxis in immunosuppressed patients: Beyond vaccination. PLoS Med.

[19] Kamar N, Abravanel F, Marion O, Couat C, Izopet J, Del Bello A (2021). Three doses of an mRNA COVID-19 vaccine in solid-organ transplant recipients. N Engl J Med.

[20] Mrak D, Tobudic S, Koblischke M (2021). SARS-CoV-2 vaccination in rituximab-treated patients: b cells promote humoral immune responses in the presence of T-cell-mediated immunity. Ann Rheum Dis.

[21] Horby P, Lim WS, Emberson JR (2021). Dexamethasone in hospitalized patients with COVID-19. N Engl J Med.

[22] Matthay MA, Thompson BT (2020). Dexamethasone in hospitalised patients with COVID-19: addressing uncertainties. Lancet Respir Med.

[23] Adler H, Ball R, Fisher M, Mortimer K, Vardhan MS (2020). Low rate of bacterial co-infection in patients with COVID-19. Lancet Microbe.

[24] Taramasso L, Sepulcri C, Mikulska M (2021). Duration of isolation and precautions in immunocompromised patients with COVID-19. J Hosp Infect.

[25] (2022). Ending isolation and precautions for people with COVID-19: interim guidance. https://www.cdc.gov/coronavirus/2019-ncov/hcp/duration-isolation.html.

